# Combined Effects of Air Pollution and Drought Stress in Tomato Landraces

**DOI:** 10.1007/s00128-026-04305-z

**Published:** 2026-07-22

**Authors:** Nora Kováts, Bettina Eck-Varanka, Katalin Hubai, József Rádi, Gábor Teke

**Affiliations:** 1 Centre for Natural Sciences, University of Pannonia, Egyetem str. 10, Veszprém, 8200 Hungary; 2 ELGOSCAR-2000 Environmental Technology and Water Management Ltd., Balatonfűzfő, 8184 Hungary

**Keywords:** Climate change, Drought stress, Atmospheric particulate matter pollution, Polycyclic aromatic hydrocarbons, Tomato landraces

## Abstract

In Europe, agriculture has been seriously affected by the increased frequency of droughts. Agricultural fields are also exposed to traffic-related air pollution. Air pollution and drought elucidate simultaneous stress, which has been scarcely studied. The major objective of the study was to evaluate the combined effects of air pollution and drought stress on different tomato varieties/landraces. 3 commercial varieties as well as 2 Hungarian landraces were selected, effects of the combined stressors were evaluated based on biomass reduction and biochemical end-points such as photosynthetic pigments concentrations and peroxidase (POD) activity. Air pollution was simulated by treating test plants according to the No. 227 OECD Guideline. Differences were experienced in the sensitivity of varieties, landraces tested: the regional landrace ‘Tápiószelei’ proved the most tolerant to any combination of treatments. Results stress the importance of the cultivation of regional landraces to cope with environmental stress in a changing climate.

**Supplementary Information:**

The online version contains supplementary material available at 10.1007/s00128-026-04305-z.

## Introduction

Drought stress is considered one of the major abiotic stresses for plants, both in anthropogenic and natural ecosystems. In Europe, increased frequency of droughts in the past two decades has been reported, seriously impacting the Central European region (Hari et al. 2020). Within the region, the Pannonian Basin is supposed to be heavily affected in the future, considering its agriculture (Crocetti et al. 2020).

Lack of appropriate water supply has considerable effects on the biochemical and physiological processes of the plants, which in turn will influence growth (Sharma et al. 2019), partially via the damage of the photosynthetic apparatus (Gong et al. 2005). Drought stress enhances the production of reactive oxygen species (ROS), resulting in oxidative stress in plant cells (Lukić et al. 2020).

Crops most possibly live in an environment where several stressors act simultaneously (Suzuki et al. 2014). In parallel with drought stress, plants can also be exposed to atmospheric pollution, especially when grown near high-trafficked sites (Hubai et al. 2024). Emission of diesel-powered vehicles is a major concern for air quality as exhaust particles bind polycyclic aromatic hydrocarbons (PAHs) and heavy metals, which might trigger reactive oxygen species (ROS) production (Velali et al. 2019).

Plants exhibit similar antioxidative responses to both drought stress and traffic-related emissions, which likely enhance each other’s effects. One major aim of the study presented here was to evaluate the effects of airborne particulate matter-bound toxic pollution and drought stress, examining these stressors individually and in combination. In order to give a quantitative estimation of potential phytotoxic responses, the No. 227 OECD Guideline for the Testing of Chemicals: Terrestrial Plant Test was followed.

For the study, tomato (*Lycopersicon esculentum* Mill.) was selected as the test species. Tomato is one of the most widely cultivated vegetables, ranking second after potato in terms of global production (EUROSTAT 2024). It has been used in experimental studies both for assessment of air pollution (e.g. Ahammed et al. 2012; Daresta et al. 2015) and for assessment of the effects of water scarcity (Lin et al. 2021).

As one of the major goals of tomato breeding is tolerance to environmental stress (Rothan et al. 2019), another important question addressed was to make a quantitative comparison of the sensitivities of different tomato varieties, including 2 regional landraces, hypothesising the better resistance of these landraces.

## Materials and Methods

### Sampling and Sample Preparation

For phytotoxicity testing, aqueous extract of diesel exhaust (PM2.5-10) was prepared (Teke et al. 2020). A 20-year-old, Euro3 light-duty vehicle was operated at idling for ×10 minutes, and particles were collected about 1 m from the tailpipe in a closed premise. A KÁLMÁN PM_2.5_ sampler was applied (flow rate 32 m^3^ h^−1^), and samples were collected on quartz filters (Whatman QMA, diameter 150 mm). The small cut filter pieces were placed in a beaker with 1000 mL high-purity water for 24 h at room temperature and stirred several times. The extract was filtered through a 0.45 μm pore size filter (GN-6 Membrane, 0.45 μm Hydrophilic mixed cellulose esters) and stored at – 20 °C until use.

### Analytical Measurements

Polyaromatic hydrocarbon concentrations in the aerosol extract were measured according to MSZ 1484-6:2003 standard (MSZ 1484-6:2003: Testing of waters. Determination of polycyclic aromatic hydrocarbons (PAH) content by gas chromatographic-mass spectrometry, LOD: 0.005 µg L^−1^ per component). Analytical measurements were carried out by the courtesy of the accredited laboratory of ELGOSCAR-2000 Environmental Technology and Water Management Ltd.

Quality assurance/quality control (QA/QC) implied that for quantification and quantifying of the sample, and for procedural recovery, internal and surrogate standards were used. Internal standard (p-Terphenyl-d14, 2-fluorobiphenyl from Restek Corporation, Bellefonte, Pennsylvania, US) and surrogate standards (Naphtalene-d8, Acenaphthene-d10, Phenanthrene-d10, Chryzene-d12 Benzo(a)pyrene-d12, and Perylene-d12, from Restek Corporation, Bellefonte, Pennsylvania, US) were used. The standards were properly diluted with GC-grade solvents (Sigma-Aldrich, St. Louis, Missouri, US) and prepared freshly before the analysis.

### Test Plants and Treatment

Five different tomato varieties/landraces have been selected (Table 1). ‘Roma’, ‘Mobil’ and ‘Lugas’ are commercially available varieties. While ‘Roma’ is an internationally available variety, available from a wide range of distributors, ‘Mobil’ and ‘Lugas’ have been selected in Hungary by ZKI (Institute for Vegetable Research - Zöldségkutató Intézet, in Hungarian). ‘Gulácsi’ and ‘Tápiószelei’ are regional landraces; their short description is also given in Table 1. ‘Roma’, ‘Mobil’ and ‘Lugas’ seeds were purchased from a commercial distributor; ‘Gulácsi’ and ‘Tápiószelei’ seeds were provided by the National Centre for Biodiversity and Gene Conservation of Tápiószele.

**Table 1:** Taxonomy and morphological description of test varieties

Variety/Landrace	Morphology
Roma	Type of growth: determinate
	Fruit form: cylindrical (egg or plum shaped)
	Average mass: 60 g
Mobil	Type of growth: indeterminate
	Fruit form: slightly flattened
	Average mass: 130 g
LugasF1	Type of growth: indeterminate
	Fruit form: cylindrical (egg or plum shaped)
	Average mass: 65 g
Tápiószelei	Type of growth: indeterminate
	Fruit form: slightly flattened
	Average mass: 46 g
Gulácsi	Type of growth: indeterminate
	Fruit form: ribbed, slightly flattened
	Average mass: 237 g

For the cultivation and treatment procedures, the protocol of the No. 227 OECD Guideline for the Testing of Chemicals: Terrestrial Plant Test (hereinafter referred to as ‘Guideline’) was followed. Seedlings were grown in 530 cm^3^ pots, which were repositioned every 2nd day to minimise environmental variability. Each pot contained one test plant. Treatments began when plants reached the 4 true leaf stage. For each variety, 4 test groups (TGs) were set where a combination of environmental stressors was applied. In each group, 10–10 containers were used. In TG1, plants received a good water supply and no PM treatment was applied, as such, this group served as control. Table 2 summarises these combinations.

**Table 2:** Description of test groups

Variety/landrace	TG1 (control)	TG2	TG3	TG4
Roma	WW/PM–	RW/PM–	WW/PM+	RW/PM+
Mobil	WW/PM–	RW/PM–	WW/PM+	RW/PM+
Lugas	WW/PM–	RW/PM–	WW/PM+	RW/PM+
Tápiószelei	WW/PM–	RW/PM–	WW/PM+	RW/PM+
Gulácsi	WW/PM–	RW/PM–	WW/PM+	RW/PM+

WW, Well-watered; RW, Reduced watering. PM–, no treatment; PM+, treatment with the PM extract

Treatment implied that the PM extract was sprayed on the above-ground parts of the tomato plants using a CONXIN Q1P-CX01-380 portable electric paint spray gun with an application volume of 5 mL/pot/treatment. Two levels of water availability were selected: high water availability (200 mL/day) (well-watered, WW) and low water availability (50 mL/day) (reduced watering, RW). These levels were set based on the study of Lin et al. (2021).

Test groups TG3 (WW/PM+) and TG4 (RW/PM+) received spraying on Day 1, Day 8 and Day 15. The first spraying was applied when seedlings reached the 4 true-leaf stage according to the Guideline. Irrigation treatments started simultaneously as the 4-leaf stage is recommended by Guida et al. (2017).

### Evaluation of Phytotoxic Effects

Phytotoxic effects were evaluated based on the following end-points: growth inhibition (biomass reduction in comparison to the control); phyotosynthetic pigments (chlorophyll a and b, and carotene), and peroxidase (POD). Photosynthetic pigments and POD were measured as described in previous papers (Hubai et al. 2021). Biomass measurements were done in 10, pigment and POD measurements in 30 replicates.

All statistical analyses were carried out using R (version 4.0.5) software. Data were analysed by Student’s* t*-test, Kruskal-Wallis chi-squared with Neményi post hoc test and two-way analysis of variance (ANOVA) with Tukey post hoc test. In each case, treatment effects were tested at 5% probability level (*P* = 0.05).

## Results

In comparison to the control (good water supply, no treatment), ‘Roma’ showed a significant decrease in biomass (ANOVA: F = 3.9009, num df = 3.000, denom df = 18.048, *p* < 0.05). Significant differences could be experienced between the WW/PM– and WW/PM+ (Tukey posthoc test: *p* < 0.01) as well as between WW/PM– and RW/PM+ groups (Tukey posthoc test: *p* < 0.05) (Fig. 1; Table 5, supplementary material). POD activity (nmol × (min × mg protein)) also showed significant differences for all individual or combined treatments (ANOVA F = 11.09, p = *p* < 0.001; Tukey posthoc test: control and RW/PM–: *p* < 0.001; control and RW/PM+: *p* < 0.05; control and WW/PM + *p* < 0.001).

Based on biomass, significant responses were found for ‘Mobil’ (ANOVA: F = 5.2354, num df = 3.00, denom df = 19.33, *p* < 0.01) (Fig. 2; Table 5, supplementary material). This variety showed significant response comparing control and RW/PM– (Tukey posthoc test: *p* = 0.0452); control and RW/PM+ (Tukey posthoc test: *p* < 0.01), and marginal non-significant response comparing control and WW/PM+ (Tukey posthoc test: *p* = 0.0509). POD activity (nmol × (min × mg protein)) also showed significant differences (ANOVA: F = 5.43, Df = 3, *p* < 0.001). In case of this variety, a significant response was found in the RW/PM- treatment group (*p* = 0.0868) and a marginally non significant response in the RW/PM+ group (*p* < 0.001).

Significant responses in biomass appeared in ‘Lugas’ (ANOVA: F = 11.429, num df = 3.000, denom df = 19.468,* p* <  0.001) (Fig. 3 and Table 5, supplementary material). This variety showed significant difference between the control and WW/PM+ (Tukey posthoc test:* p* = 0.0274); control and RW/PM– (Tukey posthoc test:* p*  < 0.01), and between control and the combination of reduced water supply and PM treatment (RW/PM+) (Tukey posthoc test: * p*  < 0.01). POD was unresponsive.

No statistically significant biomass reduction could be experienced in the two regional landraces, ‘Gulácsi’ (ANOVA: F = 1.4445, num df = 3.000, denom df = 19.966, *p* < 0.05), and ‘Tápószelei’ (ANOVA: F = 1.1342, num df = 3.0, denom df = 19.8, *p* < 0.05). POD activity (nmol × (min × mg protein)) showed significant differences in both of them: ‘Gulácsi’: ANOVA F = 10.47, *p* < 0.001; ‘Tápiószelei’: ANOVA F = 5.923, *p* < 0.001. The ‘Gulácsi’ variety showed a significant increase in the RW/PM– (Tukey posthoc test *p* < 0.001) and RW/PM+ (Tukey posthoc test *p* < 0.001) groups. For the ‘Tápiószele’ variety, a significant difference was found in the RW/PM- group (Tukey post hoc test: *p* < 0.001).

In case of photosynthetic pigments (chlorophyll-a, chlorophyll-b and carotene), non-significant differences were found in all varieties tested.

Positive responses per treatment group per variety are summarised in Table 3.

**Table 3:** Positive responses in the tested varieties

Variety/landrace	Endpoint	TG2: RW/PM–	TG3: WW/PM+	TG4: RW/PM+
Roma	Biomass	No difference	Decrease	Decrease
Mobil	Biomass	Decrease	Decrease (marginally non-significant)	Decrease
Lugas	Biomass	Decrease	Decrease	Decrease
Gulácsi	Biomass	No difference	No difference	No difference
Tápiószelei	Biomass	No difference	No difference	No difference
Roma	POD	Increase	Increase	Increase
Mobil	POD	Increase	No difference	Increase (marginally non-significant)
Lugas	POD	No difference	No difference	No difference
Gulácsi	POD	Increase	No difference	Increase
Tápiószelei	POD	Increase	No difference	No difference

WW, Well-watered; RW, Reduced watering. PM–, no treatment; PM+, treatment with the PM extract

## Discussion

The impact of drought on crop species is becoming more and more serious due to global climate change (Ma et al. 2022). It is utmostly important to understand the tolerance mechanisms of crop plants, including tomato and improve tolerant varieties (Çelik et al. 2021).

‘Roma’ showed a positive response to both PM and drought-imposed stress. POD levels increased in all treatment groups. Biomass decreased in two treatment groups: TG3 WW/PM+, indicating the deleterious effects of PM-bound chemicals and TG4 RW/PM+, indicating the combined effects of water stress and potentially toxic chemicals. These responses might demonstrate its general sensitivity to environmental stress.

It is not only a highly popular variety but has also been the subject of phytotoxicity studies. Daresta et al. (2015) used ‘Roma’ to assess the phytotoxicity of atmospheric PM, growing test plants on PM10 collected on quartz fiber filters. The resulting phytotoxic effects were directly associated with the ROS-generating behaviour of PM. ‘Roma’ was also used as a model species when the applicability of the OECD protocol was assessed for testing PM-bound phytotoxicity (Hubai et al. 2021). These studies seem to prove its proper sensitivity to phytotoxic air pollutants.

‘Mobil’, another commercial variety, also showed generally low resistance to stress. Decrease in biomass was demonstrated in RW/PM- as a response to drought stress and in RW/PM + as a response to the combination of the two stress factors. The fact that a marginally non-significant decrease was found in WW/PM+ might suggest that PM-posed chemical stress also contributes to deleterious symptoms in this variety. However, elevated levels of POD were found only in the two treatment groups, which were exposed to reduced water supply: RW/PM– and RW/PM+. As PM treatment alone did not elucidate a statistically significant response, drought seems to have a more pronounced contribution to the lower performance of this variety.

Considering the other Hungarian commercial variety, ‘Lugas’, growth impairment as expressed in reduced biomass was experienced in all treatment groups, regardless of the nature of the stress. It should also be noted that this was the only variety which did not show early signs of stress, as POD levels seemed unchanged in these groups.

Finally, for the two regional landraces, only elevated POD levels indicated toxic effects. In the case of Gulácsi, the responsive treatment groups were as follows: TG2 RW/PM– and TG4 RW/PM+; in Tápiószelei, TG2 RW/PM–. Water scarcity seems to have a negative influence even on carefully selected landraces; however, the increased levels of POD can be considered an early warning symptom (Aina et al. 2024). As no negative effect on biomass was experienced, these varieties might show good growth potential in drier climatic conditions.

Biomass reduction in test groups which received reduced water supply was found in commercial varieties. In the study of Turan et al. (2023), a significant decrease in growth was found in tomato seedlings under water deficit conditions showing measureable differences in shoot fresh and dry weight, root fresh and dry weight, plant height, and most importantly, in leaf area. Drought resistance, however, is definitely a specific feature of a given variety, in a genotype-dependent manner (Conti et al. 2019).

The inhibition of growth is a general symptom of water deficit, most frequently associated with the impact on the photosynthetic machinery and oxidative stress (Ünyayar et al. 2005). However, as no reduction of photosynthetic pigments was experienced in our study, most likely oxidative stress could be defined as the primary factor which had an inhibitory effect on growth potential.

It should be stressed that in comparison to commercial varieties, the two regional landraces showed excellent resistance. Guida et al. (2017) experimentally proved the relative drought resistance of local Italian landraces when no irrigation was applied. Landi et al. (2017) assessed the drought resistance in traditional Italian tomato landraces in comparison to commercial cultivars. Under drought stress, commercial cultivars showed the constitutive activation of the ROS scavenging machinery as a result of oxidative stress induction, while in traditional landraces, lower levels of oxidative stress were experienced. The study concluded that it could at least partly indicate a more effective ROS scavenging machinery. In general, most of the commercial cultivars can be regarded as more drought sensitive than landraces (Zdravkovic et al. 2013).

Assessing the potential phytotoxic effects of PM treatment, increased POD levels were experienced in TG3 WW/PM + and TG4 RW/PM + in the case of Roma, in TG3 WW/PM + in the case of Mobil and TG4 RW/PM + in the case of Gulácsi. A marginally significant increase was detected in TG3 WW/PM + in the case of Lugas.

In well-watered treatment groups, phytotoxic response could be associated with chemical stress. The total PAH content of the PM extract was 2.43 µg L^–1^, with the prevalence of the 3-ring fluorene (0.472 µg L^–1^) and phenanthrene (0.448 µg L^–1^) (Table 4 Supplementary material). Both PAHs mainly occur in gaseous form, but they can also be found in the particle phase (Škrdlíková et al. 2011). The sample preparation method implies that fine particles can penetrate into the extract, which also explains the presence of higher molecular weight (5–5-ring) PAHs.

Atmospheric phenanthrene showed detectable phytotoxicity on tomato in the study of Ahammed et al. (2012), and fluorene was reported to elucidate antioxidative stress in crop plants (Salehi-Lisar et al. 2015). Results given in the case of the three commercial varieties support their sensitivity to atmospheric pollution.

Comparing the sensitivity of end-points used, growth inhibition and peroxidase have proven sensitive markers in concordance with other studies (e.g. Storch-Böhm et al. 2022). In contrast to most of the results reported in the literature (Yucedag et al. 2025), phytosynthetic pigments did not show any response.

## Conclusions

Climate change is supposed to have an increasing effect on agriculture. Crops have to perform in a multistressor environment where several factors act simultaneously. In our study, the performance of selected tomato varieties was assessed in combinations of drought stress and simulated atmospheric pollution. The results of this study indicate the higher resistance of landraces toward abiotic stress, the commercial variety ‘Roma’ showing the highest, while the two regional landraces showing the lowest sensitivity. The study highlights the importance of careful selection of landraces in order to cope with environmental stress in a changing climate and to make a step towards sustainable agriculture.

## Electronic supplementary material

Below is the link to the electronic supplementary material.

Supplementary file 1 (DOCX 19 kb)

Supplementary file 2 (DOCX 19 kb)

Supplementary file 3 (DOCX 19 kb)

## References

[CR1] Ahammed GJ, Yuan HL, Ogweno JO, Zhou YH, Xia XJ (2012) Brassinosteroid alleviates phenanthrene and pyrene phytotoxicity by increasing detoxification activity and photosynthesis in tomato. Chemosphere 86:546–555. 10.1016/j.chemosphere.2011.10.038 22119279 10.1016/j.chemosphere.2011.10.038

[CR2] Aina O, Bakare OO, Fadaka AO, Keyster M, Klein A (2024) Plant biomarkers as early detection tools in stress management in food crops: a review. Planta 259(3):60. 10.1007/s00425-024-04333-1 38311674 10.1007/s00425-024-04333-1 PMC10838863

[CR3] Çelik Ö, Ayan A, Meriç S, Atak Ç (2021) Comparison of tolerance related proteomic profiles of two drought tolerant tomato mutants improved by gamma radiation. J Biotechnol 330:35–44. 10.1016/j.jbiotec.2021.02.012 33652074 10.1016/j.jbiotec.2021.02.012

[CR4] Conti V, Mareri L, Faleri C, Nepi M, Romi M et al (2019) Drought stress affects the response of italian local tomato (*Solanum lycopersicum* L.) varieties in a genotype-dependent manner. Plants 8(9):336. 10.3390/plants8090336 31500309 10.3390/plants8090336 PMC6783988

[CR5] Crocetti L, Forkel M, Fischer M, Jurečka F, Grlj A et al (2020) Earth observation for agricultural drought monitoring in the Pannonian basin (southeastern Europe): current state and future directions. Reg Environ Change 20:123. 10.1007/s10113-020-01710-w

[CR6] Daresta BE, Italiano F, de Gennaro G, Trotta M, Tutino M, Veronico P (2015) Atmospheric particulate matter (PM) effect on the growth of *Solanum lycopersicum* cv. Roma plants. Chemosphere 119:37–42. 10.1016/j.chemosphere.2014.05.054 24955951 10.1016/j.chemosphere.2014.05.054

[CR7] EUROSTAT (2024) The fruit and vegetable sector in the EU—a statistical overview. https://ec.europa.eu/eurostat/statistics-explained/index.php?title=The_fruit_and_vegetable_sector_in_the_EU_-a_statistical_overview#Data_sources, Accessed 02 01 2026

[CR8] Gong H, Zhu X, Chen K, Wang S, Zhang C (2005) Silicon alleviates oxidative damage of wheat plants in pots under drought. J Plant Sci 169:313–321. 10.1016/j.plantsci.2005.02.023

[CR9] Guida G, Sellami MH, Mistretta C, Oliva M, Buonomo R et al (2017) Agronomical, physiological and fruit quality responses of two Italian long-storage tomato landraces under rain-fed and full irrigation conditions. Agric Water Manage 180:126–135. 10.1016/j.agwat.2016.11.004

[CR10] Hari V, Rakovec O, Markonis Y, Hanel M, Kumar R (2020) Increased future occurrences of the exceptional 2018–2019 Central European drought under global warming. Sci Rep 10:12207. 10.1038/s41598-020-68872-9 32764540 10.1038/s41598-020-68872-9 PMC7413549

[CR11] Hubai K, Kováts N, Teke G (2021) Effects of urban atmospheric particulate matter on higher plants using *Lycopersicon esculentum* as model species. SN Appl Sci 3:770. 10.1007/s42452-021-04745-8

[CR12] Hubai K, Kováts N, Eck-Varanka B, Tumurbaatar S, Teke G (2024) Accumulation of atmospheric PAHs in white mustard—can the seeds be affected? Bull Environ Contam Toxicol 112(5):76. 10.1007/s00128-024-03895-w 38733550 10.1007/s00128-024-03895-w PMC11088551

[CR13] Landi S, De Lillo A, Nurcato R, Grillo S, Esposito S (2017) In-field study on traditional Italian tomato landraces: the constitutive activation of the ROS scavenging machinery reduces effects of drought stress. Plant Physiol Biochem 118:150–160. 10.1016/j.plaphy.2017.06.011 28633087 10.1016/j.plaphy.2017.06.011

[CR14] Lin PA, Paudel S, Afzal A, Shedd NL, Felton GW (2021) Changes in tolerance and resistance of a plant to insect herbivores under variable water availability. Environ Exp Bot 183:104334. 10.1016/j.envexpbot.2020.104334

[CR15] Lukić N, Kukavica B, Davidović-Plavšić B, Hasanagić D, Walter J (2020) Plant stress memory is linked to high levels of anti-oxidative enzymes over several weeks. Environ Exp Bot 178:104166. 10.1016/j.envexpbot.2020.104166

[CR16] Ma J, Tang X, Sun B, Wei J, Ma L et al (2022) A NAC transcription factor, TaNAC5D-2, acts as a positive regulator of drought tolerance through regulating water loss in wheat (*Triticum aestivum* L). Environ Exp Bot 196:104805. 10.1016/j.envexpbot.2022.104805

[CR17] Rothan C, Diouf I, Causse M (2019) Trait discovery and editing in tomato. Plant J 97:73–90. 10.1111/tpj.14152 30417464 10.1111/tpj.14152

[CR22] Škrdlíková L, Landlová L, Klánová J, Lammel G, Teke G, Hubai K, Diósi D, Kováts N (2011) Wet deposition and scavenging efficiency of gaseous and particulate phase polycyclic aromatic compounds at a central European suburban site. Atmos Environ Bull Environ Contam Tox 45(25):4305–4312. 10.1016/j.atmosenv.2011.04.072

[CR18] Salehi-Lisar SY, Deljoo S (2015) The physiological effect of fluorene on *Triticum aestivum*, *Medicago sativa*, and *Helianthus annus*. Cogent Food Agric 1:1020189. 10.1080/23311932.2015.1020189

[CR19] Sharma S, Leskovar D, Crosby K (2019) Genotypic differences in leaf gas exchange and growth responses to deficit irrigation in reticulatus and inodorus melons (*Cucumis melo* L). Photosynthetica 57:237–247. 10.32615/ps.2019.022

[CR20] Storch-Böhm RF, Somensi CA, Testolin RC, Rossa ÜB, Corrêa R et al (2022) Urban afforestation: using phytotoxicity endpoints to compare air pollution tolerance of two native Brazilian plants Aroeira (*Schinus terebinthifolius*) and Cuvatã (*Cupania vernalis*). Environ Sci Pollut Res 29:56579–56591. 10.1007/s11356-022-19890-9 10.1007/s11356-022-19890-9 35338463

[CR21] Suzuki N, Rivero RM, Shulaev V, Blumwald E, Mittler R (2014) Abiotic and biotic stress combinations. New Phytol 203:32–43. 10.1111/nph.12797 24720847 10.1111/nph.12797

[CR28] Teke G, Hubai K, Diósi D, Kováts N (2020) Assessment of foliar uptakeand accumulation of airborne polyaromatic hydrocarbons under laboratoryconditions. Bull Environ Contam Tox 104: 444-448. 10.1007/s00128-020-02814-z 10.1007/s00128-020-02814-z PMC7145778 32152686

[CR23] Turan M, Ekinci M, Argin S, Brinza M, Yildirim E (2023) Drought stress amelioration in tomato (*Solanum lycopersicum* L.) seedlings by biostimulant as regenerative agent. Front Plant Sci 14:1211210. 10.3389/fpls.2023.1211210 37662171 10.3389/fpls.2023.1211210 PMC10469020

[CR24] Ünyayar S, Keleş Y, Çekiç FÖ (2005) The antioxidative response of two tomato species with different drought tolerances as a result of drought and cadmium stress combinations. Plant Soil Environ 51(2):57–64

[CR25] Velali E, Pantazaki A, Besis A, Choli-Papadopoulou T, Samara C (2019) Oxidative stress, DNA damage, and mutagenicity induced by the extractable organic matter of airborne particulates on bacterial models. Regul Toxicol Pharm 104:59–73. 10.1016/j.yrtph.2019.03.004 10.1016/j.yrtph.2019.03.004 30872015

[CR26] Yucedag C, Yaman I, Cicek N, Zeren Cetin I (2025) Exploring the effects of vehicle-related air pollution on the photosynthetic and biochemical traits of *Cupressus macrocarpa* cv. Goldcrest. Bull Environ Contam Tox 114(5):73. 10.1007/s00128-025-04053-6 10.1007/s00128-025-04053-6 40319204

[CR27] Zdravkovic J, Jovanovic Z, Djordjevic M, Girek Z, Zdravkovic M, Stikic R (2013) Application of stress susceptibility index for drought tolerance screening of tomato populations. Genetika 45(3):679–689. 10.2298/GENSR1303679Z

