# Supplementary material for: Combined Effects of Air Pollution and Drought Stress in Tomato Landraces
Source: Bull Environ Contam Toxicol. 2026 Jul 22;117(2):33. doi: 10.1007/s00128-026-04305-z (PMC13391766; doi:10.1007/s00128-026-04305-z)
Supplement: Supplementary file 1 — (DOCX 19 kb) [file 128_2026_4305_MOESM1_ESM.docx]

**Fig.1** Biomass changes in ’Roma’ in comparison to the control. 10 replicates were used for the measurements.

**Fig.2** Biomass changes in ’Mobil’ in comparison to the control. 10 replicates were used for the measurements.

**Fig.3** Biomass changes in ’Lugas’ in comparison to the control. 10 replicates were used for the measurements.
