# Supplementary material for: Combined Effects of Air Pollution and Drought Stress in Tomato Landraces
Source: Bull Environ Contam Toxicol. 2026 Jul 22;117(2):33. doi: 10.1007/s00128-026-04305-z (PMC13391766; doi:10.1007/s00128-026-04305-z)
Supplement: Supplementary file 2 — (DOCX 19 kb) [file 128_2026_4305_MOESM2_ESM.docx]

**Table 4** Concentration of PAHs in the aerosol extract

| PAH |  | Concentration  [µg/l] |
| --- | --- | --- |
| two rings | Naphthalene | 0.001 |
|  | 2-methyl-naphthalene | 0.394 |
|  | 1-methyl-naphthalene | 0.367 |
| three rings | Acenaphthylene | 0.12 |
|  | Acenaphthene | 0.052 |
|  | Fluorene | 0.472 |
|  | Phenanthrene | 0.448 |
|  | Anthracene | 0.058 |
| four rings | Fluoranthene | 0.074 |
|  | Pyrene | 0.081 |
|  | Benzanthracene | 0.032 |
|  | Chrysene | 0.013 |
| five rings | Benzo(b)fluoranthene | 0.064 |
|  | Benzo(k)fluoranthene | 0.036 |
|  | Benzo(e)pyrene | 0.055 |
|  | Benzo(a)pyrene | 0.043 |
|  | Dibenzo(a.h)anthracene | 0.045 |
| six rings | Indeno1.2.3CD-Pyrene | 0.033 |
|  | Benzo(g.h.i)perylene | 0.035 |
| Total PAHs |  | 2.43 |
