# Supplementary material for: Combined Effects of Air Pollution and Drought Stress in Tomato Landraces
Source: Bull Environ Contam Toxicol. 2026 Jul 22;117(2):33. doi: 10.1007/s00128-026-04305-z (PMC13391766; doi:10.1007/s00128-026-04305-z)
Supplement: Supplementary file 3 — (DOCX 19 kb) [file 128_2026_4305_MOESM3_ESM.docx]

**Table 5.** Biomasss changes data in different treatment groups (fresh biomass in g). WW/PM- (well watered, no chemical treatment) group served as control. In each group, 10-10 replicates were used.

| **Treatment** | **Lugas** | **Mobil** | **Roma** |
| --- | --- | --- | --- |
| WW/PM- | 3.530 | 2.554 | 2.268 |
| WW/PM- | 3.407 | 2.453 | 1.524 |
| WW/PM- | 3.213 | 3.676 | 1.815 |
| WW/PM- | 4.237 | 3.36 | 1.554 |
| WW/PM- | 3.363 | 2.426 | 1.753 |
| WW/PM- | 2.117 | 3.329 | 1.715 |
| WW/PM- | 4.351 | 2.973 | 1.764 |
| WW/PM- | 3.895 | 2.882 | 2.036 |
| WW/PM- | 3.505 | 2.725 | 3.279 |
| WW/PM- | 2.913 | 2.713 | 0.969 |
| RW/PM- | 0.3 | 2.690 | 0.877 |
| RW/PM- | 2.4 | 2.223 | 1.023 |
| RW/PM- | 1.9 | 2.745 | 1.303 |
| RW/PM- | 2.0 | 2.205 | 1.222 |
| RW/PM- | 2.8 | 3.052 | 2.06 |
| RW/PM- | 1.2 | 2.378 | 1.783 |
| RW/PM- | 3.0 | 1.926 | 1.771 |
| RW/PM- | 1.1 | 2.592 | 1.422 |
| RW/PM- | 3.8 | 2.472 | NA |
| RW/PM- | 3.2 | 1.631 | NA |
| WW/PM+ | 2.551 | 2.539 | 0.99 |
| WW/PM+ | 1.292 | 2.990 | 0.492 |
| WW/PM+ | 2.278 | 2.160 | 1.831 |
| WW/PM+ | 2.803 | 2.835 | 1.25 |
| WW/PM+ | 1.177 | 2.380 | 1.915 |
| WW/PM+ | 0.937 | 2.677 | 2.15 |
| WW/PM+ | 4.039 | 1.503 | 0.825 |
| WW/PM+ | 2.755 | 2.296 | 0.652 |
| WW/PM+ | 2.755 | 2.449 | 0.773 |
| WW/PM+ | 2.87 | 2.191 | 1.491 |
| RW/PM+ | 1.616 | 2.376 | 0.74 |
| RW/PM+ | 2.937 | 2.140 | 0.649 |
| RW/PM+ | 1.875 | 1.392 | 1.218 |
| RW/PM+ | 0.942 | 2.122 | 1.581 |
| RW/PM+ | 1.985 | 2.979 | 1.259 |
| RW/PM+ | 1.017 | 1.842 | 0.828 |
| RW/PM+ | 2.150 | 2.098 | 1.011 |
| RW/PM+ | 2.033 | 2.348 | 1.068 |
| RW/PM+ | 1.815 | 1.906 | 1.606 |
| RW/PM+ | 1.948 | NA | NA |
